# Survivorship Care for People With Cancer in the Indo-Pacific: The Imperative to Harness Political Determinants, International Exchange, and Technological Innovation

**DOI:** 10.1200/GO.23.00052

**Published:** 2023-06-08

**Authors:** Edward Christopher Dee, Frances Dominique V. Ho, Kaisin Yee, Vivian K. Lin

**Affiliations:** Edward Christopher Dee, MD, Department of Radiation Oncology, Memorial Sloan Kettering Cancer Center, New York, NY, USA; Frances Dominique V. Ho, BSc, College of Medicine, University of the Philippines, Manila, Philippines; Kaisin Yee, BSc, Department of Global Health and Population, Harvard T.H. Chan School of Public Health, Boston, MA, USA, SingHealth Duke-NUS Global Health Institute, Singapore, Singapore; and Vivian K. Lin, DrPH, MPH, LKS Faculty of Medicine, University of Hong Kong, Hong Kong, China

## TO THE EDITOR:

The Indo-Pacific, a region home to 60% of the world's population, is marked by vast diversity in geography, language, culture, history, economic, and geopolitical context.^1^ Approximately 40% of the world's cancer survivors, individuals who have received a cancer diagnosis, reside in this region.^1^ In the companion to this article, Koczwara et al^1^ reviewed care for cancer survivors, illustrated problems of access, and suggested steps to advance access. We write to highlight (1) the need to consider the political determinants of health, (2) the imperative to collaborate within and across borders, and (3) the role of innovation and technology in promoting access to cancer survivorship care in the Indo-Pacific.

The political context that surrounds health care is critical to consider and leverage in the provision of survivorship care across the cancer continuum.^2^ In the Philippines, for example, strong political will galvanized the passage of the National Integrated Cancer Control Act (NICCA) in 2019, which laid out an agenda that prominently included the provision of survivorship care.^3^ It is hoped that with the change of administration in 2022, further political will would galvanize equitable implementation of the NICCA, in light of the gravity of the need and general support from the medical community.^4^ In Thailand, the rising mortality attributed to cancer since the early 2000s prompted the development of the National Cancer Control Program, leading to the establishment of multiple regional cancer hospitals that provide streamlined services for patients across the continuum of cancer care.^5^ In both the Philippines and Thailand, universal health coverage provided the foundation for cancer care, but the passage of the Universal Health Care Bill in the Philippines and the 30-Baht Scheme in Thailand similarly required political will.^6,7^ Even with these policy foundations, building of multidisciplinary teams and strengthening primary health care, along with the financing for chronic care and access to essential medicines, remain a work in progress. These examples demonstrate that political forces—often catalyzed by stakeholders including patient advocates and providers—can not only effect tangible change but also may take time and strong advocacy coalitions. Thus, to improve access to survivorship care, policymakers should leverage these political determinants, integrating the perspectives of patients, providers, and other stakeholders.

Koczwara et al^1^ highlighted parallel themes in the barriers different countries face in providing cancer survivorship care. These include heterogeneous and great patient needs, limitations in health system capacity and coordination, and segregated survivorship research programs. Stakeholders who aim to improve survivorship care can make use of the world's increasing globalization through digital health to overcome these challenges, particularly through multinational collaboration.^8,9^ The authors recommended leveraging high-level platforms such as the WHO-hosted Asia Pacific Observatory on Health System and Policies to advance collaboration, but research networks may have limited impact in moving policy action forward. Beyond this, however, institutional collaboratives, such as global health institutes and alliances, can set focused agendas around cancer survivorship care and facilitate work across a broad base of academic research, policy studies, clinical and administrative capacity building, and innovation. Furthermore, although there are regional opportunities to advance cancer survivorship care, local coordination must not be neglected. Research collaboration networks must engage with policy makers in order for system change to occur in a timely and appropriate fashion, and these can occur through ongoing policy dialogues or engagement through regional intergovernmental fora, such as the Association of Southeast Asian Nations or Pacific Islands Forum. Collaboration across sectors locally can streamline limited resources, reduce duplicative and fragmented services, and improve patient experience in navigating the health and supportive care system. In Singapore, survivor-centric models that encourage institutional collaboration between academic medical centers, primary care physicians, and community patient support organizations have been identified as imperative to coordinated cancer survivorship care.^10^ Early efforts have been made to build partnerships across professional roles.^11^

Finally, we suggest the inclusion of technology and innovation as a component in the authors' map of the inter-relationship between care delivery, data monitoring, and research in cancer survivorship. The diverse country contexts in the Indo-Pacific make the region particularly fertile for the development of both advanced and frugal innovation. In the archipelagos of Indonesia and the Philippines, high mobile connectivity has bolstered the adoption of telehealth, particularly during the COVID-19 pandemic. These systems have the potential to be adapted and adopted for use in cancer survivorship care. In service innovation, best practices in established cancer centers can be spread through bilateral or regional collaborations.^12^ For example, Indonesia has recently established several bilateral partnerships between the Dharmais National Cancer Center, their national referral center, and academic and clinical institutes in India, including cancer centers such as the Tata Memorial Hospital.^13,14^ In the low- and middle-income countries in the region, as well as the small island developing states, digital health and m-health can offer the best opportunity to increase access to quality specialist services while supporting patients and families in health literacy and decision making; these efforts must be coupled with investment in the information and communication technology infrastructure so as not to exacerbate the digital divide for small countries such as the Pacific Islands and Timor Leste. Notably, technology is rarely one-size fits-all, particularly in the diverse context of the Indo-Pacific countries.

Perhaps less common is the role of artificial intelligence (AI)–based technologies in scaling access to care in both the acute term and the long term.^15^ The challenges associated with care provision in settings with low numbers of trained professionals may be mitigated by technology such as AI-based cancer screening, imaging analysis, and wearable devices.^16-18^ Oral cancer image analysis in a rural Indian setting is one such example,^19^ as has more general cancer diagnostics in China, in an effort to bridge rural hospitals with specialist centers.^20^ Although technology advances are by no means a panacea, careful and equitable implementation of these advances may improve access to care for patients in low-resource settings.

We echo the commentary by Koczwara et al^1^ that the unique and complex contexts of each Indo-Pacific country can and should be balanced with opportunities to foster regional and international collaboration. Additionally, we propose the consideration of political determinants of health, increased coordination at both local and regional levels, and leveraging on innovation and technology to improve access to cancer survivorship in the Indo-Pacific region.
